# RH5.1-CyRPA-Ripr antigen combination vaccine shows little improvement over RH5.1 in a preclinical setting

**DOI:** 10.3389/fcimb.2022.1049065

**Published:** 2022-12-20

**Authors:** Julie Healer, Jennifer K. Thompson, Karen L. Mackwell, Cecille D. Browne, Benjamin A. Seager, Anna Ngo, Kym N. Lowes, Sarah E. Silk, David Pulido, Lloyd D. W. King, Jayne M. Christen, Amy R. Noe, Vinayaka Kotraiah, Paul J. Masendycz, Rajkannan Rajagopalan, Leanne Lucas, Marianne M. Stanford, Lorraine Soisson, Carter Diggs, Robin Miller, Susan Youll, Kaye Wycherley, Simon J. Draper, Alan F. Cowman

**Affiliations:** ^1^ The Walter and Eliza Hall Institute of Medical Research, Parkville, VIC, Australia; ^2^ University of Melbourne, Melbourne, VIC, Australia; ^3^ Leidos Life Sciences, Frederick, MD, United States; ^4^ Department of Biochemistry, University of Oxford, Oxford, United Kingdom; ^5^ IMV, Inc., Dartmouth, NS, Canada; ^6^ Malaria Vaccine Development Program, United States Agency for International Development (USAID), Washington, DC, United States

**Keywords:** malaria, *Plasmodium falciparum*, RH5 complex, vaccine, merozoite invasion, Ripr, CyRPA

## Abstract

**Background:**

RH5 is the leading vaccine candidate for the Plasmodium falciparum blood stage and has shown impact on parasite growth in the blood in a human clinical trial. RH5 binds to Ripr and CyRPA at the apical end of the invasive merozoite form, and this complex, designated RCR, is essential for entry into human erythrocytes. RH5 has advanced to human clinical trials, and the impact on parasite growth in the blood was encouraging but modest. This study assessed the potential of a protein-in-adjuvant blood stage malaria vaccine based on a combination of RH5, Ripr and CyRPA to provide improved neutralizing activity against P. falciparum in vitro.

**Methods:**

Mice were immunized with the individual RCR antigens to down select the best performing adjuvant formulation and rats were immunized with the individual RCR antigens to select the correct antigen dose. A second cohort of rats were immunized with single, double and triple antigen combinations to assess immunogenicity and parasite neutralizing activity in growth inhibition assays.

**Results:**

The DPX® platform was identified as the best performing formulation in potentiating P. falciparum inhibitory antibody responses to these antigens. The three antigens derived from RH5, Ripr and CyRPA proteins formulated with DPX induced highly inhibitory parasite neutralising antibodies. Notably, RH5 either as a single antigen or in combination with Ripr and/or CyRPA, induced inhibitory antibodies that outperformed CyRPA, Ripr.

**Conclusion:**

An RCR combination vaccine may not induce substantially improved protective immunity as compared with RH5 as a single immunogen in a clinical setting and leaves the development pathway open for other antigens to be combined with RH5 as a next generation malaria vaccine.

## Introduction

Dramatic reductions in the incidence and numbers of deaths attributable to malaria have been accomplished since the implementation of coordinated vector control and drug administration campaigns began in earnest around 20 years ago, with 21 countries achieving elimination or reaching zero transmission within that time (WHO, 2020). These public health successes have so far been achievable without a vaccine; however, progress has stagnated, and it is a widely held belief that for many highly endemic regions, malaria control and elimination will not be possible without a highly effective vaccine.

The most well-developed malaria vaccine against *P. falciparum*, the causative agent of the most severe form of malaria in humans, is RTS,S/AS01, (Stoute et al., 1998) which in October 2021 was recommended for use in children under the age of five years old living in regions with moderate to high malaria endemicity in Africa. The immune responses engendered by this vaccine target the circumsporozoite (CSP) antigen on the surface of the sporozoite – a parasite stage that is injected by a blood feeding mosquito. The RTS,S/AS01 vaccine is moderately efficacious in preventing clinical malaria but is shown to have limited durability with respect to the protective immune response in the target population (Alonso et al., 2004; Alonso et al., 2005; Campo et al., 2014). While a huge step forward, this falls short of the World Health Organisation recommendation for the vaccine roadmap of 75% efficacy over 2 years (https://www.who.int/publications/m/item/malaria-vaccine-technology-roadmap) (MalERA Consultative Group on Vaccines, 2011). A second-generation vaccine is needed and would ideally target different stages of the parasite life cycle exposed to the human immune system, including the blood stages, given this part of the life cycle is responsible for the pathogenic features of malarial disease.

Identification and characterisation of antigens that are essential to parasite survival has revealed a protein complex that is required for merozoite invasion of human erythrocytes (Hayton et al., 2008; Baum et al., 2009; Douglas et al., 2011; Dreyer et al., 2012; Bustamante et al., 2013; Reddy et al., 2014; Douglas et al., 2015; Reddy et al., 2015; Volz et al., 2016). This complex is comprised of three essential proteins, RH5, CyRPA and Ripr, which form a heterotrimeric complex (RCR) (Chen et al., 2011; Reddy et al., 2015; Volz et al., 2016; Wong et al., 2019) at the merozoite apical tip where RH5 binds to the erythrocyte receptor basigin in a critical step early in the invasion process (Crosnier et al., 2011; Weiss et al., 2015; Volz et al., 2016). Rabbit antibodies specific for RH5, CyRPA or Ripr can inhibit invasion of *P. falciparum* merozoites *in vitro* (Hayton et al., 2008; Baum et al., 2009; Chen et al., 2011; Dreyer et al., 2012; Bustamante et al., 2013; Reddy et al., 2014; Azasi et al., 2020). Invasion inhibitory domains and epitopes have been identified for each protein and in some cases mouse or human monoclonal antibodies can block assembly of this complex (Healer et al., 2019) or direct interaction of RH5 with its receptor basigin (Wright et al., 2014; Healer et al., 2019; Alanine et al., 2019). Furthermore, RH5 antibodies have been shown to provide synergistic inhibition with antibodies directed against other *P. falciparum* invasion proteins, including CyRPA (Illingworth et al., 2019; Alanine et al., 2019; Ragotte et al., 2022). These studies have suggested that a combination of RH5, CyRPA and Ripr as a vaccine may provide an additive, or perhaps even synergistic, effect in terms of their ability to induce antibodies that block RCR complex formation and binding to the basigin receptor and consequently *P. falciparum* merozoite invasion. Indeed, a combination of antibodies against RH5, CyRPA or Ripr has been tested in several studies, with varying degrees of synergy observed in their ability to inhibit *P. falciparum* growth (Bustamante et al., 2013; Reddy et al., 2014; Favuzza et al., 2017; Healer et al., 2019; Illingworth et al., 2019; Azasi et al., 2020; Ragotte et al., 2022).

The three components of the RCR complex have been individually assessed for their suitability as potential vaccine candidates (Bustamante et al., 2013; Reddy et al., 2014; Wright et al., 2014; Ntege et al., 2016; Chen et al., 2017), but at the time of writing, only the first identified member of the RCR complex, RH5, has progressed into clinical trials (Payne et al., 2017; Jin et al., 2018; Minassian et al., 2021). Results of a Phase I/IIa trial of RH5.1/AS01_B_, a full-length RH5 protein-in-adjuvant formulation, has shown this vaccine to be safe and well-tolerated. Using a novel vaccination regimen of a fractional third dose 6 months following the first immunisation, RH5/AS01_B_ induced antibody titers that were maintained out to ~2 years after the primary immunisation (Payne et al., 2017). This study also substantiated earlier results of RH5 vaccination in animal models that found a strong correlation between RH5 antibody titers, *in vitro* growth-inhibition activity (GIA), and a reduction in the parasite multiplication rate *in vivo* upon *P. falciparum* challenge (Douglas et al., 2015). While this effect was modest, it does raise the possibility that potentiating the RH5 response may improve the reduction in parasite growth rate to a point that is clinically efficacious.

This study was undertaken to determine if vaccination of rats with a combination of antigens representing the identified members of the RCR complex was superior to RH5 alone in inducing a polyclonal antibody response that blocked growth of *P. falciparum*, and to evaluate different adjuvants that may be used for progressing new formulations to human clinical trials.

## Materials and methods

### Study designs

**Figure 1:**
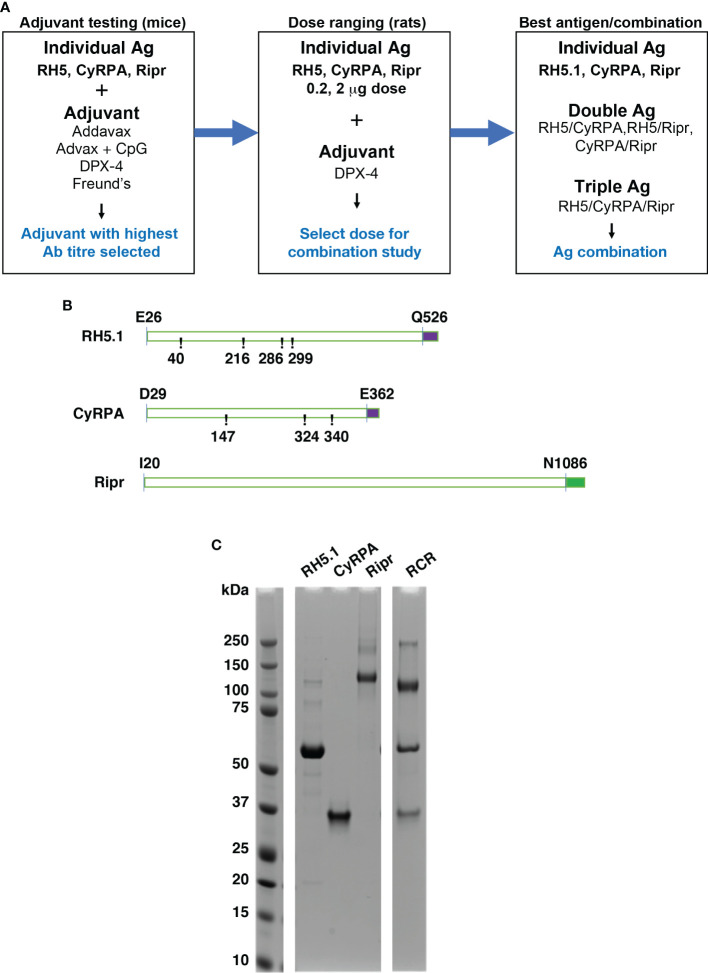
Strategy and antigens in the RCR complex used in single, double, and triple combinations to determine immunogenicity and ability to block *P. falciparum* growth. **(A)** Workflow of experiments to determine immunogenicity and development of growth inhibitory antibodies for the RCR complex. Adjuvants and antigen dose were determined prior to immunisation experiments using combinations of RH5.1, CyRPA and Ripr. **(B)** Schematic of recombinantly expressed proteins expressed with C-terminal affinity tags. RH5.1 (RH5) and CyRPA were expressed with a C-tag (EPEA) (purple) and Ripr was expressed with a StrepII tag (green) for purification. CyRPA and RH5 sequences were modified from the 3D7 wildtype sequence (at numbered positions) to remove potential N-linked glycosylation sites. RH5.1 and Ripr were expressed in S2 *Drosophila* cells, whereas CyRPA was expressed in mammalian HEK293 cells. **(C)** RH5.1, CyRPA and Ripr and RCR complex purified antigens analysed by SDS-PAGE and Coomassie staining to visualize the bands.

The objectives of the *in vivo* studies were to select a clinically approved adjuvant for use in subsequent studies, perform dose ranging assessments, and perform a comparative assessment of individual and combinations of RCR antigens in a rodent vaccination model (**Figures 1A, B**). Entire coding regions minus the signal peptides of the antigens were selected for expression. RH5.1 (E26-Q526) (Jin et al., 2018) and Ripr (I20-N1086) were expressed using ExpreS^2^ion Biotechnologies *Drosophila* S2 cell platform (Hjerrild et al., 2016), while CyRPA (D29-E362) was expressed from mammalian HEK293 cells. In all cases, the *P. falciparum* 3D7 parasite sequence was used to design the coding regions of each protein. RH5.1 (T40A, T216A, T286A and T299A) and CyRPA (S147A, T324A and T340A) were modified from the corresponding 3D7 protein sequence of each to remove potential N-linked glycosylation sites. Potential N-linked glycosylation sites were removed to increase yield of protein expression and to block potential effects on immunogenicity such as epitope masking. Ripr was expressed for immunisation with no sequence modifications. For production of RCR complex for use in ELISA, Ripr was expressed with the following amino acid substitutions (N103Q, N144Q, N228Q, N334Q, N480Q, N498Q, N506Q, N526Q, N646Q, N647Q, N964Q and N1021Q) that were identified as potential glycosylation sites (Azasi et al., 2020) and the RCR complex was made by mixing RH5.1, CyRPA and Ripr in the designated ratios.

For the triple antigen combinations, antigens were pre-complexed in either equimolar (1:1:1) ratio i.e., dose amounts (µg) based on 14 pmole per antigen; the total protein dose per animal was 3.14 µg. MW of RH5.1, CyRPA and Ripr are 60,219, 39,470 and 124,840 g/mole, respectively, or mass (1:1:1) ratio with a final protein dose per animal of 3 µg (**Table 1**).

**Table 1:** Single protein and RCR antigen combination dose assignments.

Cohort	Formulation	RH5.1, µg	CyRPA, µg	Ripr, µg
1	RH5.1 + DPX	1	0	0
2	CyRPA + DPX	0	1	0
3	Ripr + DPX	0	0	1
4	RH5.1 + Ripr + DPX	1	0	1
5	RH5.1 + CyRPA + DPX	1	1	0
6	Ripr + CyRPA +DPX	0	1	1
* 7	RH5.1 + Ripr + CyRPA + DPX (Equimolar Ratio)	0.84*	0.55*	1.75*
8	RH5.1 + Ripr + CyRPA + DPX (Mass)	1	1	1
9	Adjuvant Alone (DPX; Negative Control)	0	0	0

*Cohort #7 dose amounts (µg) are based on 14 pmole per antigen; the total protein dose per animal in Cohort #7 was 3.14 µg. MW of RH5.1, CyRPA and Ripr are 60,219, 39,470 and 124,840 g/mole, respectively.

In cohorts 7 & 8, antigen combinations were in RCR complex form prior to formulation with DPX.

### Animal immunisation

*Adjuvant scouting study:* Six 8-week-old female BALB/c mice (WEHI Kew Animal Facility) per cohort were injected intramuscularly (IM) with 20 µg antigen + adjuvant in 100 µL volumes on days 0 and 28. Four adjuvants were assessed in this study, Freund’s incomplete adjuvant (Sigma Aldrich), Addavax (*In vivo*Gen), Advax + CpG (Vaxine), and the DPX^®^ platform (manufactured by IMV Inc.), a non-aqueous, versatile, lipid-in-oil drug delivery technology that educates a specific, targeted and, long-lasting immune responses; DPX was packed with the antigen(s) of interest, immunomodulator Pam3Cys and freeze-dried; and then reconstituted in Montanide ISA 51 VG prior to injections. Pre-bleed samples were taken on day -2, and mice were terminally bled by cardiac puncture on day 42 for serum preparation. Sera from the six mice per cohort were pooled for analysis.

*Dose ranging and combination studies:* DPX-formulated antigens were supplied as lyophilized material in individual vials along with the oil diluent (Montanide ISA 51 VG; Seppic) and 1 mL Medallion syringes (Merit Medical System). and the reconstitution was performed at WEHI as per manufacturer’s instructions. Six 8-week-old female Wistar rats (Animal Resource Centre, Western Australia) per cohort were injected intramuscularly into alternative hind legs for each immunization on days 0, 28 and 56. Blood samples for serum preparation were taken on days -2, 42 and 70. Rat sera were analysed individually.

### Serum and IgG preparation

Serum was prepared by centrifugation from clotted whole blood and stored at -20 °C until use.

Immunoglobulin G was purified on Protein A Sepharose and buffer exchanged into PBS then concentrated to 20 mg/mL using Amicon Ultra centrifugal filters (Millipore).

### ELISA

Nunc Immuno ELISA plates (Fisher) were coated with antigens diluted in Na_2_CO_3_ buffer pH 9.6 at 1 mg/mL and dispensed at 50 μL per well. Plates were incubated at 4°C overnight followed by a wash step. Test sera were diluted threefold from 1/100 – 1/18x10^6^ in PBS and plates incubated at room temperature for 90 min. Following a wash step, 50 µL of HRP conjugated anti-mouse or rat Ig pre-diluted to 1 in 1000 in PBS was added to each well and plates incubated at room temperature for 60 min. Following a wash step, 50 µL of TMB substrate was added to all wells and incubated for 5 min. 30 µL of 2 M Sulphuric acid was added to stop the development reaction. Plates were read at 450 nm on ELISA plate reader. OD1 titer interpolations were performed using R Studio software and statistical analysis performed using Prism software.

### Growth inhibition activity assays

Growth Inhibition Activity (GIA) assays were set up for a one growth cycle assay. 10 µL of synchronized trophozoite stage 3D7 *P. falciparum* parasites were added to 35 µL of O+ erythrocytes at 2% hematocrit in 96 well round bottom microtiter plates (Falcon) at 0.5% parasitemia. Then 5 µL of IgG at 20, 10, 5 or 2.5 mg/mL added to each well resulting in a final IgG concentration of 2, 1, 0.5 or 0.25 mg/mL per well. Following parasite growth for a period of 42-48 hr, culture plates were then frozen at -80°C overnight. LDH activity in trophozoite stage parasites was quantified with 3-acetylpyridine adenine dinucleotide (APAD) by measuring absorbance of nitro blue tetrazolium (NBT) at 650 nm. Plates were thawed at room temperature for < 4 h prior to LDH measurement as follows: 45 mL of fresh LDH reaction mix (174 mM sodium L-lactate, 214 mM 3-acetyl pyridine adenine dinucleotide (APAD), 270 mM Nitro Blue tetrazolium chloride (NBT), 4.35 U/mL diaphorase, 0.7% Tween 20, 100mM Tris-HCl pH 7.5) was dispensed into each well using a Multidrop combi dispenser at high-speed ensuring good mixing. Absorbance at 650 nm was measured in an EnVision (PerkinElmer) plate reader after 20 min of incubation at room temperature. Data were analysed using Dotmatics 5.3 and Spotfire 7.11.1 (Tibco) software.

GIA with the purified rat IgG was expressed as percent inhibition calculated using the formula: 100 − [(OD650 of infected RBCs with test IgG − OD650 of uninfected RBCs only)/(OD650 of infected RBCs with Non-immune Rat IgG − OD650 of uninfected RBCs only) × 100].

### GIA assay reversal

Antigens were bound to affinity resin depending on their respective affinity tags. RH5.1, CyRPA and the RCR complex were bound to Capture Select C-tag affinity matrix (Thermo Fisher) and Ripr bound to StrepTactin resin (Sigma Aldrich). 50 µL of resin pre-washed in PBS was incubated with 50 µg of antigen in 200 µL PBS and incubated in individual 0.8 mL micro-biospin columns (Biorad), rotating, overnight at 4 °C. Flow-through was collected by pulse spin in benchtop centrifuge and resin washed 3 times with 1 mL PBS to remove unbound antigen. 200 µL of cohort IgG minipools at 20 mg/mL were incubated for 30 min at room temperature with antigen-captured resin, followed by pulse-spin to collect flow-through. This process was repeated twice with the IgG flow-through. This contained the unbound (depleted) IgG and was retained. The depleted IgG was sterile filtered by centrifugation (Costar) and used in the GIA reversal experiments.

## Results

The series of studies reported herein were designed to assess the potential of a protein-in-adjuvant blood stage malaria vaccine based on the essential protein complex RCR that is required for *P. falciparum* merozoite invasion of erythrocytes (Volz et al., 2016). Due to the encouraging results from RH5.1 clinical trials, we investigated whether the combination of members of the RCR complex would induce higher levels of growth-inhibitory activity upon vaccination of rats. This study explores the potential of the RCR antigen combination as a next-generation blood stage combination vaccine for clinical testing to investigate whether the addition of the other members of this essential protein complex, CyRPA and Ripr, induced higher levels of growth-inhibitory activity upon vaccination of rats and, if so, whether the RCR antigen combination could be a next-generation blood stage combination vaccine for clinical testing. To do this, RH5.1, CyRPA and Ripr antigens were formulated for immunization with drug delivery platforms approved for use in humans that included Addavax, Advax+CpG, and DPX, in addition to Freund’s incomplete adjuvant as a control adjuvant. The aim was to select the best formulation with respect to the immune response and ability of the antibodies to inhibit growth of *P. falciparum in vitro* (**Figure 1A**).

The 3D7 *P. falciparum* sequence was used for designing recombinant proteins with RH5.1 and Ripr expressed in *Drosophila* S2 cells and CyRPA in HEK293 cells. RH5.1 and CyRPA included mutations to remove potential N-linked glycosylation sites (**Figure 1B**). The three proteins were purified by affinity chromatography using C-terminal affinity tags (**Figures 1B, C**). As shown in **Figure 1C**, the three individual antigens used for immunization are relatively pure, aggregates and breakdown products are present as minor contaminants, with RH5.1 migrating at 60 kDa, CyRPA at approximately 37 kDa and Ripr at 120 kDa. The interactions in the RCR complex are non-covalent and all three components migrate as single bands under reducing SDS-PAGE conditions and can form a complex of 480 kDa (**Supplementary Figure 4**).

### DPX-formulated RH5.1, CyRPA and Ripr induce high-titer, *P. falciparum* neutralising antibodies

Preclinical studies investigating immunogenicity of vaccine candidate antigens in animal models routinely use Freund’s adjuvant as an immune potentiator since it yields high titer antibody responses that are desirable for downstream functional analyses. Freund’s adjuvant, however, is not appropriate for clinical use in humans but was used here to compare antibody responses generated with other adjuvants. In this study, drug delivery platforms have been evaluated against Freund’s incomplete adjuvant, Addavax, Advax + CpG and DPX^®^ were compared in parallel for their ability to generate high titer IgG responses in mice when formulated with recombinant domains of the antigens RH5.1, CyRPA and Ripr (**Table S1**). In this experiment, mice were immunised on day zero and 28 with individual antigens formulated with the above adjuvants at a dose of 20 μg per immunisation (**Table S1**). To assess comparative immunogenicity, sera from 6 mice per immunisation group were pooled and tested in ELISA. Antibody titers in sera from mice immunised with antigens formulated with DPX were generally higher overall, indicating that DPX was the superior immunogen delivery vehicle (**Table S2** and **Figure S1**). In this drug delivery platform scouting comparison, DPX performed best and consequently was chosen for further studies.

**Figure 2:**
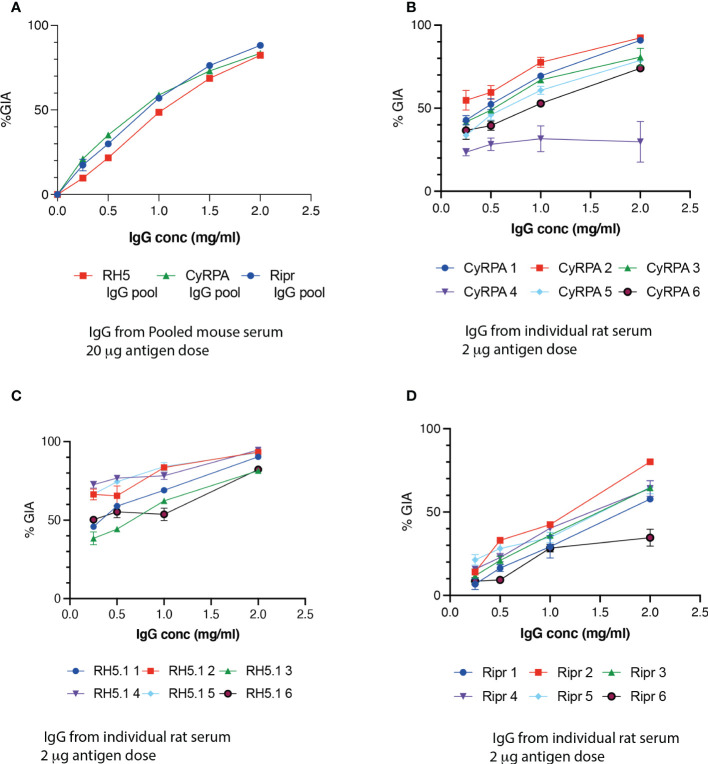
DPX-formulated candidates induce *P. falciparum* growth inhibitory antibodies against RH5, CyRPA and Ripr in mice and rats. **(A)** A GIA assay was performed with IgG pooled from 6 mice immunized with 20 μg antigen formulated with DPX - RH5 (red squares), CyRPA (green triangles) or Ripr (blue circles). Intra-cohort variability in GIA of IgG from six individual rats immunized with 2 μg antigen - RH5.1 **(B)** CyRPA **(C)** and Ripr **(D)** antigens were formulated with DPX. Error bars represent SEM of triplicate wells.

To determine if antibodies generated in the mice immunised with DPX + RH5.1, CyRPA or Ripr were able to inhibit growth of *P. falciparum*, growth inhibition activity (GIA) assays were performed. IgG purified from serum pools was highly inhibitory in GIA assays (**Figure 2A**). In this experiment, IgG reduced parasite growth in a dose-dependent manner, and at the highest IgG concentration of 2 mg/mL, inhibition values ranged from 82% (RH5.1 and CyRPA) to 88% (Ripr). A 2-way ANOVA revealed a statistically significant difference between antibodies across the dilution range of the experiment (F = 51.3, *p <*0.001).

To optimize antibody responses with the RH5.1, CyPRA and Ripr vaccination combinations, a dose-ranging experiment was next conducted in the Wistar rat model by immunizing with individual proteins formulated in DPX (**Table S3**). The rat model allowed for analysis of individual animal responses, due to a higher serum volume recovery at terminal bleed. There was a dose dependency showing higher antibody titers induced when using 2 μg compared with 0.2 μg for RH5.1, CyRPA and Ripr (**Figure S2**). Notably, the 20 μg dose of RH5.1 induced lower antibody titers than the 2 μg dose. The 20 μg dose was administered for the RH5.1 group only and was included as a control to bridge from the adjuvant-selection experiment. Boosting of antibody titers between the second and third immunization was seen in cohorts 4 and 6 that received 2 μg of CyRPA and Ripr, respectively (**Tables S3** and **Figure S2**), whereas no boosting was evident in groups receiving RH5.1 at the higher dosage of 2 or 20 μg. This result indicates that at a vaccination dose of 2 μg, a two-dose regimen would be sufficient for RH5.1, whereas three doses were optimal for CyRPA and Ripr. Consistent with findings from the previous experiment in mice, for the DPX cohorts, antibody titers were highest in the Ripr cohorts followed by RH5.1 and CyRPA.

Functional activity as measured by GIA with IgG purified from individual rats immunized with 2 μg antigen per dose over 3 immunizations (**Figures 2B–D**) was comparable at the highest IgG concentration with that of IgG pools from the initial 2-dose immunizations with 20 μg in mice (**Figure 2A**) at the highest IgG concentration of 2 mg/ml, confirming that an immunization dose of 20 μg was excessive for these antigens. The highest overall GIA was observed in the RH5.1 group (**Figure 2B**), with a high GIA (average 57%; range 46-72%) even at the lowest IgG concentration tested (0.25 mg/mL). GIA in the CyRPA group was like that of RH5 (**Figure 2C**), whereas IgG from rats immunized with Ripr resulted in the lowest GIA of the three antigens (**Figure 2D**), with negligible activity at the lowest IgG concentration. A possible explanation for the higher GIA activity at lower IgG concentrations for CyRPA and RH5 observed for the rat IgG compared with mouse is that the three-dose regimen induces a higher functional antibody response.

The immunogenicity and GIA against *P. falciparum* obtained by comparing the different drug delivery platforms and dosages was used to determine a final antigen dose for RH5.1, CyRPA, Ripr and the combinations of these antigens (**Table 1**). All subsequent experiments were performed with DPX using an antigen dose of 1-3 μg depending on whether they were for single, double or triple antigen vaccinations. In the triple antigen combinations, RCR antigen complexes were pre-formed prior to formulation with DPX.

### RCR antigen combinations elicit high-titer, *P. falciparum* growth inhibitory antibodies

**Figure 3:**
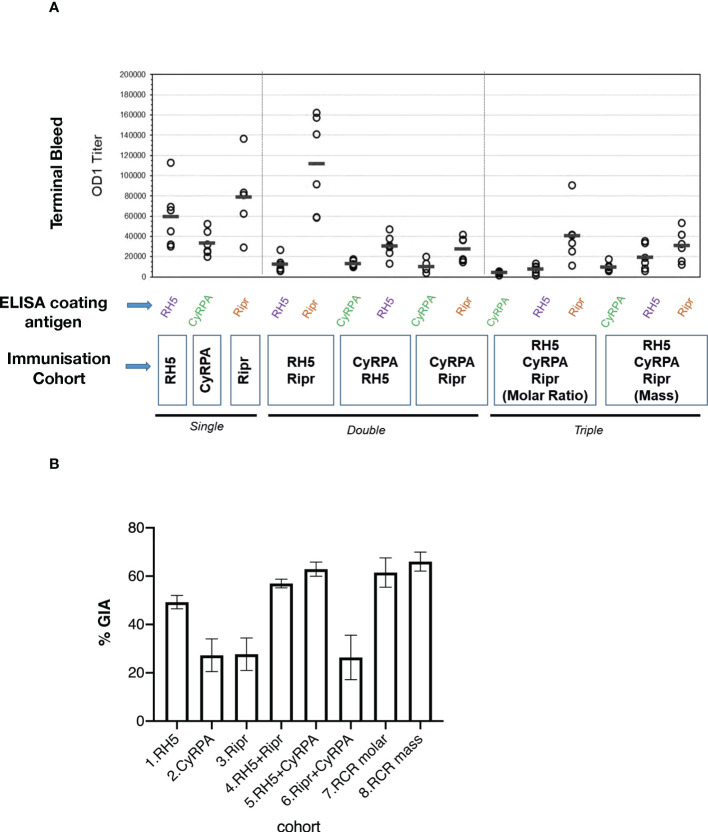
Functional activity is not correlated with immunogenicity in RCR multiple antigen combination immunisations. **(A)** Antibody reactivity in terminal bleed sera from individual rats was tested in ELISA against individual antigen components and the results are shown as OD1 titers calculated from 4PL curve fits based on the dilution curves of individual samples. Circles represent the OD1 titer from an individual rat serum titration and bars show the median of the six serum OD1 values. **(B)** GIA in IgG purified from individual rat sera. Bars show mean % GIA in each cohort of 6 rats at an IgG concentration of 2 mg/mL. Error bars show SEM.

Determination of antibody titers by ELISA in sera from individual rats immunised with either single, double, or triple antigen combinations (**Table 1**) showed that, overall, higher antibody titers were elicited by antigens immunised as single immunogens (**Figure 3A**). This suggests that there may be some antigenic competition that limits the immune response to each antigen when delivered in combination. The exception to this was the response to the RH5.1+Ripr double antigen combination, where the anti-Ripr antibody titer was significantly higher than that against RH5.1 (t-test, two-tailed, t=5.016, DF=10 p=0.0005), and was the highest titer generated in the entire immunisation experiment (**Figure 3A**). Of the three immunisations with individual antigens, the lowest terminal bleed OD1 titer was against CyRPA. These results are consistent with Ripr being more immunogenic than RH5.1 or CyRPA.

To determine if antibody titers correlated with the ability to block growth of *P. falciparum*, GIA of the IgG purified from individual rats was measured (**Figure 3B**). The highest GIA was observed in the triple antigen complex RCR cohorts, and the RH5.1+CyRPA immunised cohort (**Figure 3B** and **Figure S3**). For the individual antigen vaccinated cohorts, RH5.1 induced significantly higher GIA than either CyRPA or Ripr. Overall, the GIA was highest in the immunised cohorts that included RH5.1. Pairwise statistical comparison indicated that there was no significant difference in GIA between the RCR equimolar cohort and any of the RH5.1-containing cohorts, including RH5.1 alone at the highest IgG concentration of 2 mg/ml (**Table S4**). These data suggest that RH5.1 is the most effective antigen in raising invasion blocking antibodies and that the addition of other antigens within the RCR complex (as formulated and dosed in this study) does not provide significant additional inhibitory activity. However, GIA activity was higher with RCR IgG compared with RH5 IgG when all concentrations are analysed (**Figure S3** and **Table S5**).

**Figure 4:**
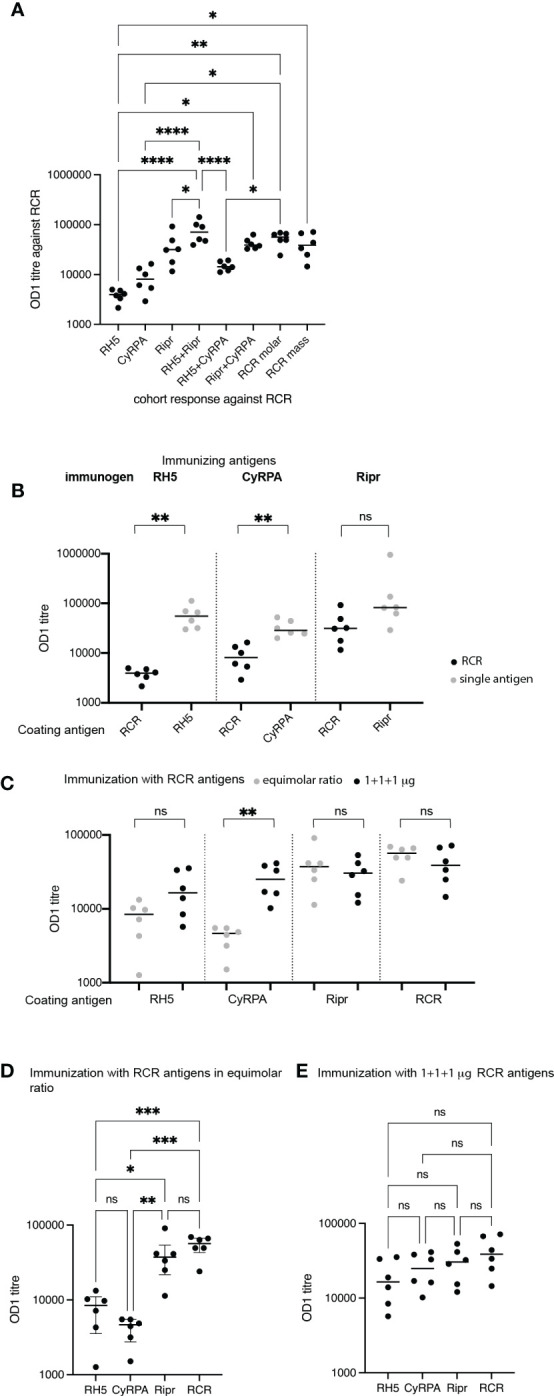
Antibody titers reveal differences in responses to individual and RCR-complexed antigens. **(A)** Antibody responses against the RCR complex in sera from RH5.1, CyRPA, Ripr, RH5.1+Ripr, RH5.1+CyRPA, Ripr+CyRPA, RCR (equimolar ratio) and RCR (mass ratio) immunized rats. The immunogens are shown on the x-axis, and OD1 titers of each cohort against the RCR complex are shown on the y-axis. Only significant associations are shown. **** p<0.0001, ** p<0.005, * p<0.05. **(B)** Antibody titers in rats immunized with single antigens RH5.1, CyRPA and Ripr are shown against the RCR complex (in black circles) and the immunogen (in grey circles). Circles show individual serum OD1 values and group medians shown by lines. Significant differences between cohort responses to the single antigen and RCR calculated using Mann-Whitney U test with Prism 9 software to calculate *p* values. ns, not significant; ** *p* = 0.01. **(C)** Immunization with RCR antigens in a 1:1:1 mass ratio induced significantly higher anti-CyRPA titers than the equimolar ratio immunization. RCR equimolar responses are shown in grey circles and RCR mass responses in black circles. Circles show individual serum OD1 values and group means are shown by lines. Data was tested for normality and significant differences between groups was tested by unpaired t-test ** p<0.005. **(D)** Pairwise comparison of antibody titers against RH5, CyRPA, Ripr and RCR in cohorts immunized with RCR in an equimolar ratio. *** p<0.001 **(E)** Pairwise comparison of antibody titers against RH5.1, CyRPA, Ripr and RCR in cohorts immunized with RCR in a mass ratio. For **(A, D, E)**: Circles show individual serum OD1 values and group means are shown by lines. Data was tested for normality and testing for significant differences between groups was conducted by one way ANOVA with Tukey’s test for multiple comparisons using Prism software to calculate *p* values.

To determine if vaccination with protein combinations limits antibody development to epitopes at the protein-protein interacting domains, antibody titers were measured against pre-complexed RCR as coating antigen. Combination immunisation cohorts had high titer antibody responses to the RCR complex, as did the cohort receiving Ripr as a single antigen whereas RH5.1 and CyRPA cohorts showed the lowest OD1 titers against the RCR complex. Inter-cohort comparison of antibody titers showed that in all three cohorts immunized with a single antigen, reactivity to the single antigen was higher than to the RCR complex (**Figure 4B**).

The highest titers were observed in the cohort immunized with RH5.1+Ripr (**Figure 4A**).

To ascertain whether there were qualitative or quantitative differences resulting from immunisation with RCR antigens delivered in an equimolar ratio or in a 1:1:1 mass ratio, we compared responses to individual antigens and the RCR complex in these cohorts. Antibody titers to CyRPA were higher when the antigens were administered in a 1:1:1 mass ratio, compared with the equimolar ratio regimen, although this difference did not reach statistical significance (**Figure 4C**). The lower dose of CyRPA in the equimolar regimen is suboptimal compared with the even higher dosing of RH5.1 and Ripr.

As expected, the RCR combination immunizations elicited the highest antibody titers to the RCR complex as compared with the single antigens, although these were not significantly higher than reactivity against Ripr as a single antigen. Responses to both RH5.1 and CyRPA were significantly lower than those to Ripr and RCR in those animals immunised with RCR in an equimolar ratio (**Figure 4D**). This contrasted with responses elicited by immunisation with RCR antigens in a mass ratio where no significant differences between reactivity to RH5.1, CyRPA, Ripr or RCR were seen (**Figure 4E**).

### Antibodies targeting Ripr contribute less than those targeting RH5 or CyRPA to the parasite neutralising activity of RCR-elicited responses

**Figure 5:**
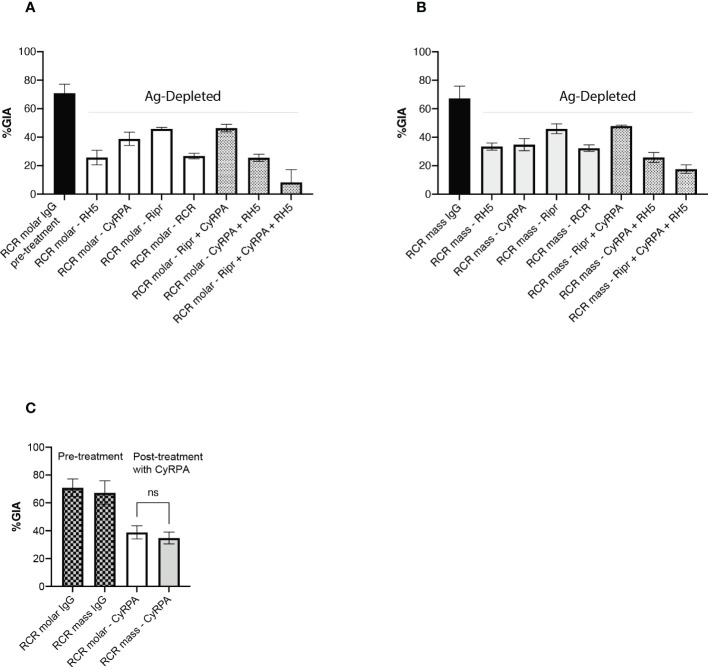
Depletion of specific antibodies to determine effect on inhibition of *P. falciparum* growth. IgG pools were made from the RCR equimolar antigen cohort IgG **(A)** and RCR mass cohort IgG **(B)** and incubated with antigens to deplete specific antibodies. Black bars - before depletion with antigen and others after depletion with specific antigen as indicated. Open bars – single antigen treatment (RCR complex is counted here as a single antigen). Hatched bars - antigen combinations. **(C)** Data extracted from panels **(A**, **B)** showing that GIA of RCR equimolar IgG and RCR mass IgG following CyRPA-depletion are not significantly different from each other. **(A–C)** Histograms show mean ± SD of two independent experiments (each performed in triplicate wells). ns, not significant.

GIA-reversal experiments in which antigen-specific IgG were depleted by incubation of the IgG with antigen bound to Sepharose beads, were performed to assess the relative contribution to functional GIA of antibodies targeting individual antigens within the sera from rats vaccinated with RCR combinations (**Figure 5**). This was of particular interest in relation to anti-CyRPA responses since the two different RCR immunization regimens elicited significantly different antibody titers against CyRPA (**Figure 4C**). Contrasting with the RCR ELISA results, the GIA reversal experiment indicated that functional antibodies targeting CyRPA were of a similar parasite-neutralising potency in GIA assays in the two cohorts (**Figure 5C**). In the RCR equimolar-elicited responses, antibodies targeting RH5 were the predominant contributors to the parasite neutralising activity (**Figure 5A**), whereas neutralising activity was similar for antibodies targeting RH5 and CyRPA in the RCR mass cohort (**Figure 5B**). Interestingly, the RCR antigen complex did not completely reverse parasite neutralising activity in either immunisation cohort, whereas a near complete reversal of GIA was observed when RCR equimolar IgG was preincubated with a mixture of the three antigens (**Figure 5A**).

## Discussion

The prospect of delivering a clinically efficacious malaria vaccine that targets the highly replicative blood stage of *P. falciparum* has been boosted recently with the publication of results from a Phase I/IIa clinical trial of RH5.1 formulated in AS01_B_ showing a good safety and tolerability profile in naïve UK-based adults and importantly, a significant reduction in parasite growth rate after blood stage challenge (Minassian et al., 2021). A possible route to improved efficacy of an RH5-based vaccine is to include other antigens in combination with RH5 (Azasi et al., 2020). We chose to explore an immunisation approach using the natural partners complexed with RH5 in *P. falciparum*, CyRPA and Ripr (Chen et al., 2011; Reddy et al., 2015; Volz et al., 2016), which have both independently shown positive results in preclinical studies (Bustamante et al., 2013; Wright et al., 2014; Reddy et al., 2014; Ntege et al., 2016; Chen et al., 2017; Favuzza et al., 2017; Healer et al., 2019; Illingworth et al., 2019; Azasi et al., 2020).

A novel formulation emerged as the best performing of the adjuvant platforms tested alongside Freund’s incomplete adjuvant. DPX is a non-aqueous, versatile, lipid-in-oil drug delivery platform, which can incorporate an array of bioactive molecules including peptides, proteins, small molecules, nucleic acids, and virus-like particles to create long-lasting, targeted immune responses to mitigate disease with limited adverse events. DPX-formulations, maveropepimut-S (formerly DPX-Survivac) and DPX-RSV have been evaluated in previous clinical studies in cancer and infectious disease, respectively (Berinstein et al., 2015; Langley et al., 2018) and were safe and well-tolerated.

In this study, the DPX platform has been formulated with RCR antigens to enhance antibody responses. Antigens were formulated into lipid vesicles, lyophilized, and reconstituted directly into Montanide ISA 51 VG prior to immunization.

A rat model was developed to assess individual variation in responsiveness within a vaccination cohort. This was tested in an antigen dose study and proved useful in allowing a blood draw volume of approximately 10 mL from cardiac puncture per animal, which provided adequate volumes of serum for downstream applications, including affinity purification of IgG for multiple GIA assays. The dose-ranging study determined that for RH5, the higher antigen dose of 20 μg resulted in a lower antibody titer than the lower dose of 2 μg. This finding was corroborated in the GIA assay results with IgG purified from these sera, indicating that the immune system was saturated above a certain level of antigen.

The combination study was designed to directly compare immunisation outcomes between cohorts vaccinated with single, double, and triple antigen combinations. In a previous study, we showed that combining RH5, CyRPA and Ripr recombinant proteins together in an equimolar ratio led to the spontaneous assembly of the RCR complex (Wong et al., 2019). In this study, another RCR vaccination cohort was tested where 1 μg each of the three antigens were combined (RCR mass) prior to formulation with DPX. This was included as a bridge to the other cohorts where 1 μg of the individual antigens was delivered per dose. The only significant difference observed between responses of the two RCR vaccine cohorts was the higher antibody titer to CyRPA induced by the RCR mass immunisation, explainable by the higher antigen dose for this particular antigen used in this vaccine cohort. This did not, however, affect either the overall GIA or the CyRPA-attributable GIA of the RCR equimolar immunization cohort, as this was not significantly different from GIA of the RCR mass cohort.

The RCR complex extracted from the DPX formulation post lyophilization showed the presence of both RCR complex and individual proteins with some interference of the DPX formulation materials in gel electrophoresis (results not shown). It was possible, however, that a proportion of the antigens are not in complex, and it would have to be assumed that even if some complex was delivered during immunization, a significant amount of the combination antigen dose would have been individual uncomplexed proteins. The GIA reversal studies suggested that RCR did not remain in a complex following formulation or immunization since GIA reversal with the intact RCR complex was significantly less than the level of reversal observed when the antigens were not in complex. This was observed regardless of whether the RCR complex was delivered in an equimolar or 1:1:1 μg regime.

The main finding from the combination immunization study was that a sizeable proportion of the functional GIA induced by RCR vaccination was attributable to antibodies targeting RH5. In the GIA reversal study, RH5 antibody depletion resulted in the largest reduction in GIA. This finding may appear to conflict with findings from the drug delivery platforms selection study in mice, where a much higher dose of antigen (20 μg) was used. In that study, responses against Ripr and CyRPA were as potent as those against RH5. However, this finding suggests that among the three antigens, a much higher dose of Ripr was required to generate a similar functional response. In two independent, successive studies, generation of inhibitory monoclonal antibodies against Ripr have both identified a single neutralising epitope within the EGF 7 domain in the C-terminal region of the protein (Healer et al., 2019). So, despite its larger size, (the molecular weight of Ripr is more than double that of RH5 and three times that of CyRPA), the number of neutralizing epitopes on Ripr is potentially relatively small compared with RH5 and CyRPA.

In terms of antibody titer, immunodominance of Ripr over RH5 and CyRPA was clear in all combinations where Ripr was present. This is likely to be due to its relatively larger size, which presents many more epitopes to the immune system.

It is worth noting that in the RH5.1/AS01_B_ clinical trial, as well as there being a strong correlation between IgG1 level and GIA, the top-ranking correlate in the delay to diagnosis was the IgA1 response whose mode of action may be independent of GIA and associated with an Fc-receptor mediated effect *via* neutrophil phagocytosis (Minassian et al., 2021). The current study was not designed to test non-GIA immune effector mechanisms of parasite inhibition.

It was interesting that there was a lack of correlation between total antibody titer and the GIA of the IgG antibodies. This would suggest that there are a limited number of inhibitory epitopes within the RH5, CyRPA and Ripr proteins and that most antibodies to other epitopes do not interfere with the function of the complex. This appears to be the case for Ripr as only a single inhibitory epitope has been identified so far (Healer et al., 2019). An inhibitory epitope has been identified for CyRPA (Favuzza et al., 2017; Chen et al., 2017; Healer et al., 2019; Ragotte et al., 2022) and in one study, the anti-CyRPA monoclonal antibody 8A7 blocked the interaction of CyRPA with RH5 (Healer et al., 2019). The inhibitory monoclonal antibodies raised in mice against RH5 mostly cause blockade of basigin binding but one has been identified that blocks interaction with CyRPA (Healer et al., 2019).

A significant finding was that there is no evidence of synergistic GIA in a co-immunization setting and that the findings of additive or synergistic activity between monoclonal antibodies targeting RH5 and CyRPA (Ragotte et al., 2022) and other merozoite antigens (Alanine et al., 2019; Azasi et al., 2020) may not be relevant to a polyclonal response following vaccination. The challenge now is to use the information gained from such studies to design vaccines that efficiently direct high titer responses to inhibitory epitopes leading to a sustained response required to protect against the highly replicative and persistent blood stages of *P. falciparum*.

In summary, all three components of the RCR protein complex could induce parasite neutralizing antibodies, however RH5.1, either as a single antigen or in combination with CyRPA and/or Ripr, was superior to CyRPA or Ripr. Although immunization with the RCR antigen combinations provided similar levels of neutralising antibodies as RH5.1 alone at high IgG concentrations, there was no significant improvement in GIA with RCR over RH5.1 alone. These findings would not warrant further investment in an RCR based vaccine over RH5.1. As RH5.1 vaccine development vaccine is already well advanced, it continues to be the favoured blood stage component of a combination vaccine. This leaves the development pathway open for other candidate antigens, possibly targeting different lifecycle stages, to be combined with RH5 in a next generation malaria vaccine.

## Data availability statement

The original contributions presented in the study are included in the article/**Supplementary Material**. Further inquiries can be directed to the corresponding author.

## Ethics statement

The animal study was reviewed and approved by The Walter and Eliza Hall Institute of Medical Research Animal Ethics Committee.

## Author contributions

JH - performed parasite GIA and data analysis, and writing the manuscript. KM, PM and KW – animal immunisations. JH, JT, KM and KW - Ripr and CyRPA polyclonal and monoclonal antibody development and screening. KM, PM, and KW – ELISA assays. BS – RCR complex formation and analysis. AN – performed GIA LDH assay. LL, RR – supplied DPX-related constituents, performed antigen formulation, CB – project management and data analysis (Leidos). JC, AN, VK– project management (Leidos), LS, CD, RM, SY – project management (USAID), RCR complex formation (IMV). SS, DP, LK, SD – supplied RCR, RH5.1 and CyRPA for dose ranging and combination immunization studies. AC and JH were responsible for overall project strategy and management, data interpretation and writing the manuscript. Ripr for immunization was procured from ExpreS^2^ion Biotechnologies, Denmark. All authors contributed to the article and approved the submitted version.
