# Supplementary material for: RH5.1-CyRPA-Ripr antigen combination vaccine shows little improvement over RH5.1 in a preclinical setting
Source: Front Cell Infect Microbiol. 2022 Dec 20;12:1049065. doi: 10.3389/fcimb.2022.1049065 (PMC9807911; doi:10.3389/fcimb.2022.1049065)
Supplement: Supplementary file 1 [file DataSheet_1.pdf]

Supplementary Material

A

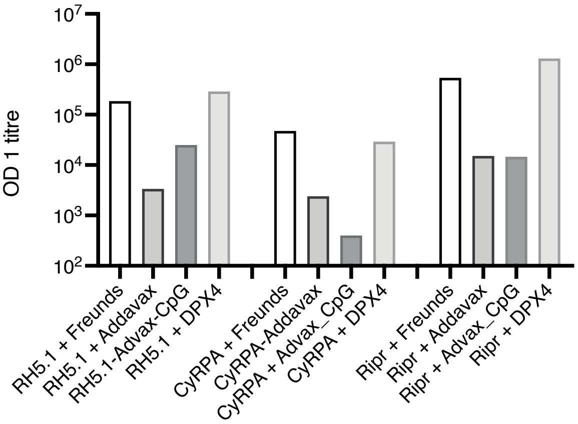

B

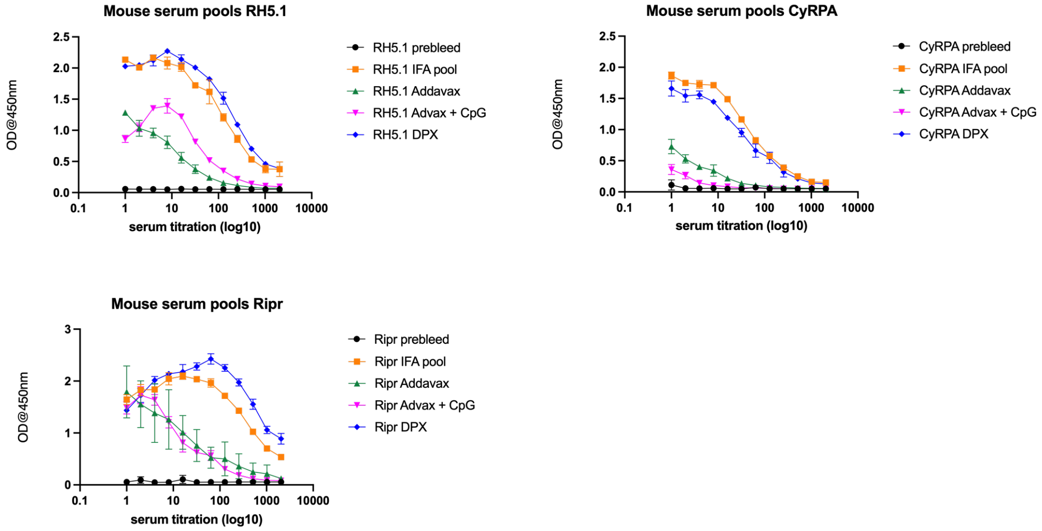

Supplementary Figure 1. Adjuvant scouting experiment - DPX-4 formulations induced high antibody titres after two immunisations. A. Histogram shows comparative OD1 titres in pooled mouse terminal bleed sera. OD1 titers were determined using 4PL Curve fit based on average of absorbances per duplicate per dilution per cohort. B. Panels show full ELISA titration for pooled terminal bleed sera. Points shown are mean ± SD of duplicate wells.

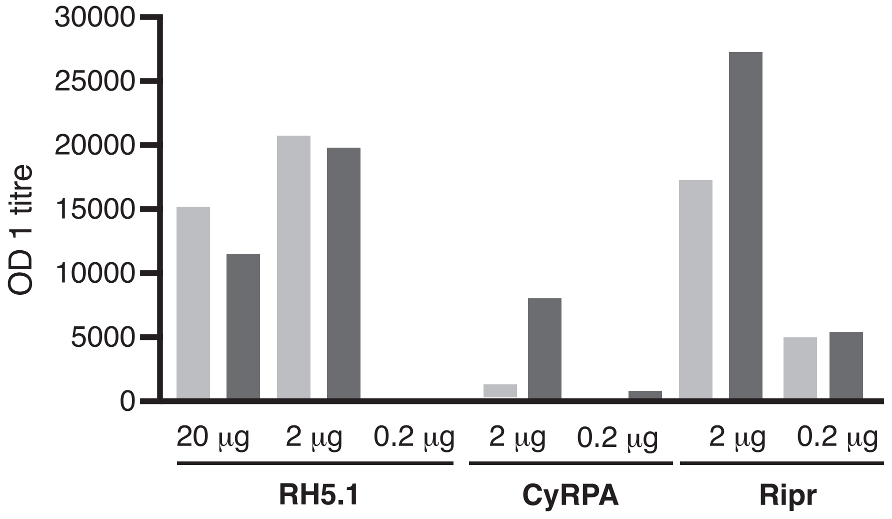

Supplementary Figure 2. Dose differentiation between 2 and 0.2 µg dose for all three antigens Rat Test Bleed (Day 42) and Terminal Bleed (Day 70) ELISA Titres (OD=1) are shown as a histogram. Bars show the OD 1 titer from 6 individual rat serum titers per cohort. Light grey and dark grey bars show result for test and terminal bleed respectively for each cohort. OD1 titres were determined using 4PL Curve fit based on average of duplicate absorbances per dilution per cohort of 6 individual sera.

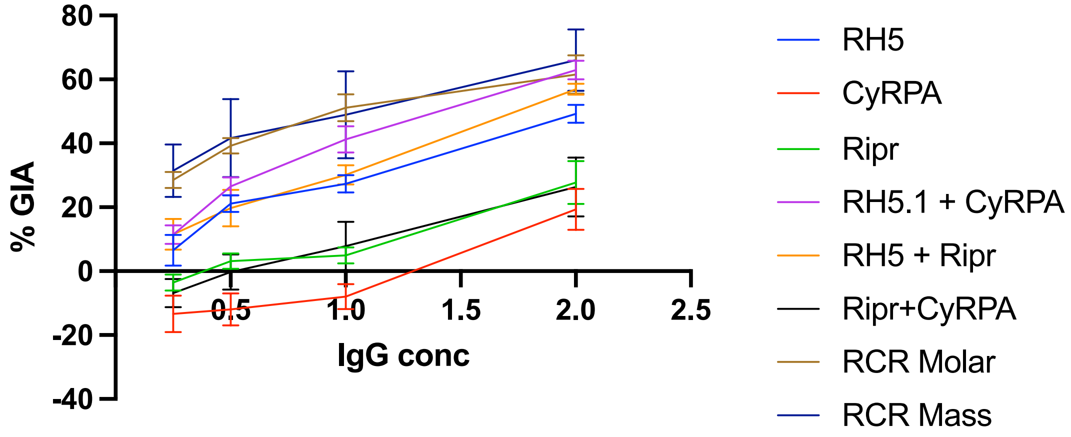

Supplementary Figure 3. Growth inhibition assays (GIA) for anti-sera from individual rats vaccinated with individual and combination antigens. Lines show mean ± SEM % GIA of IgG from 6 individual rats for anti-RH5.1, anti-CyRPA, anti-Ripr, anti-RH5.1+Ripr, anti-RH5.1+CyRPA, anti-Ripr+CyRPA, anti-RCR molar (equimolar mixture of

RH5.1+Ripr+CyRPA) and anti-RCR mass, (equivalent amount by mass of RH5.1+Ripr+CyRPA). Negative GIA occurs when the control IgG (non-immune rat IgG) has a slight growth inhibitory effect. Data was not corrected for this.

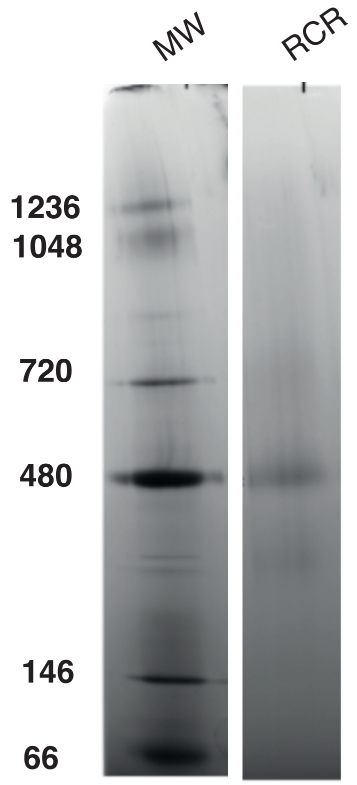

Supplementary Figure 4. RH5 + CyRPA + Ripr RCR complex. The RCR complex runs at approximately 480Kda. 2 μg of the RCR complex was run on native PAGE

Table S1 relates to supplementary figure 1 and shows the study outline of the adjuvant down selection experiment in mice.

Study 1 – Adjuvant Scouting Study

Cohort	Formulation	R	# Mice, strain	Study Day			
				-2	0	28	42
1	RIPR (20 μg) + IFA	IM	6, BALB/c		√	√	
2	RH5.1 (20 μg) + IFA	IM	6, BALB/c		√	√	
3	CyRPA (20 μg) + IFA	IM	6, BALB/c		√	√	
4	RIPR (20 μg) + AddaVax	IM	6, BALB/c	Pre-bleed	√	√	Terminal bleed
5	RH5.1 (20 μg) + AddaVax	IM	6, BALB/c		√	√	
6	CyRPA (20 μg) + AddaVax	IM	6, BALB/c		√	√	
7	RIPR (20 μg) + Advax+CpG	IM	6, BALB/c		√	√	
8	RH5.1 (20 μg) + Advax+CpG	IM	6, BALB/c		√	√	

9	CyRPA (20 μg) + Advax+CpG	IM	6, BALB/c		√	√	
10	RIPR (20 μg) + DPX4	IM	6, BALB/c		√	√	
11	RH5.1 (20 μg) + DPX4	IM	6, BALB/c		√	√	
12	CyRPA (20 μg) + DPX4	IM	6, BALB/c		√	√	

Table S2 Adjuvant Scouting experiment. Responses against the immunizing antigen in serum pools from 6 mice immunized with IFA, Addavax, Advax+CpG or DPX4 were compared using a one-way ANOVA with Tukey's test for multiple comparisons. **** denotes p<0.0001, *** p<0.001, ** p<0.005, NS not significant.

Adjuvant	RH5.1	CyRPA	Ripr
IFA v DPX	NS	NS	NS
Advax+CpG v DPX	**	**	****
Addavax v DPX	****	***	****
Advax+CpG v Addavax	NS	NS	NS

Table S3 relates to supplementary figure 2 and shows the study outline for the antigen dose ranging study in rats. Immunizations were performed on days 0, 28 and 56 as shown by ticks.

Study 2 – Dose-Ranging Study

Cohort	Formulation	R	# Rats, strain	Study Day					
				-2	0	28	42	56	70
1	RH5.1 protein, 20 μg + DPX4	IM	6, Wistar	Pre-bleed	√	√	Test bleed	√	Terminal Bleed
2	RH5.1 protein, 2 μg + DPX4	IM	6, Wistar		√	√		√	
3	RH5.1 protein, 0.2 μg + DPX4	IM	6, Wistar		√	√		√	
4	CyRPA protein, 2 μg + DPX4	IM	6, Wistar		√	√		√	
5	CyRPA protein, 0.2 μg + DPX4	IM	6, Wistar		√	√		√	
6	RIPR protein, 2 μg + DPX4	IM	6, Wistar		√	√		√	
7	RIPR protein, 0.2 μg + DPX4	IM	6, Wistar		√	√		√	
8	Adjuvant alone (Negative Control)	IM	6, Wistar		√	√		√	

Table S4 relates to Fig. 3B and shows pairwise comparisons of the mean GIA values for each cohort at IgG concentration of 2 mg/ml for each antigen cohort.

Cohort # / Antigen	2 CyRPA	3 Ripr	4 RH5 + Ripr	5 RH5 + CyRPA	6 Ripr + CyRPA	7 RCR Molar	8 RCR Mass
1 RH5	NS	NS	NS	NS	NS	NS	NS
2 CyRPA		NS	*	**	NS	**	***
3 Ripr			*	**	NS	**	***
4 RH5 + Ripr				NS	**	NS	NS
5 RH5 + CyRPA					***	NS	NS
6 Ripr + CyRPA						**	***
7 RCR Molar							NS

Significance testing was conducted by the ordinary one-way ANOVA with Tukey's test for multiple comparisons using Prism 9 software to calculate p values. NS = not significant; * p<0.05, **p<0.01, *** p<0.001.

Table S5 relates to supplementary figure 3 and shows pairwise comparisons of the mean GIA values for all IgG concentrations for each antigen cohort.

Cohort # / Antigen	2 CyRPA	3 Ripr	4 RH5 + Ripr	5 RH5 + CyRPA	6 Ripr + CyRPA	7 RCR Molar	8 RCR Mass
1 RH5	****	****	NS	NS	NS	****	****
2 CyRPA		*	****	****	****	****	****
3 Ripr			****	****	****	****	****
4 RH5 + Ripr				NS	NS	**	***
5 RH5 + CyRPA					NS	NS	*
6 Ripr + CyRPA						**	***
7 RCR Molar							NS

Significance testing was conducted by the ordinary two-way ANOVA with Tukey's test for multiple comparisons using Prism 9 software to calculate p values. NS = not significant; * p<0.05, **p<0.01, *** p<0.001, **** p<0.0001
